# 三叶青多糖增强Lewis肺癌小鼠抗肿瘤免疫反应

**DOI:** 10.3779/j.issn.1009-3419.2023.106.16

**Published:** 2023-08-20

**Authors:** Wenju ZHAO, Yong ZHU, Zhengxue LU

**Affiliations:** 401220 重庆，重庆市长寿区人民医院肿瘤内科; Department of Oncology, Changshou District People's Hospital, Chongqing 401220, China

**Keywords:** 三叶青多糖, 信号调节蛋白α, 分化抗原簇47, 肺肿瘤, 免疫反应, Radix tetrastigme polysaccharide, Signal regulatory protein α, Cluster of differentiation 47, Lung neoplasms, Immune response

## Abstract

**背景与目的:**

肺癌具有高致病率和高致死率，但肺癌治疗仍缺乏低毒高效的抗肿瘤药物。三叶青多糖在抗肿瘤治疗方面具有开发价值。本文旨在观察三叶青多糖对Lewis肺癌小鼠免疫反应的影响，并探讨其分子机制。

**方法:**

建立Lewis肺癌小鼠模型并采用随机数字表法进行分组。脾多肽组灌胃50 mg/kg脾多肽，三叶青多糖低、中、高剂量组分别灌胃62.5、125、250 mg/kg三叶青多糖，模型组和对照组灌胃等容积生理盐水。比较肿瘤形成、转移情况；苏木素-伊红（haematoxylin-eosin, HE）染色观察肿瘤细胞病理学变化；流式细胞术检测巨噬细胞吞噬能力、凋亡水平、M1/M2型极化水平及外周血T细胞亚群和细胞因子水平；噻唑蓝溴化四唑（methyl thiazolyldiphenyl tetrazolium, MTT）法检测巨噬细胞增殖活性；β-D-吡喃半乳糖苷氯酚红（chlorophenol red-β-D-galactopyranoside, CPRG）法检测树突状细胞（dendritic cell, DC）抗原提呈功能；实时荧光定量聚合酶链反应（real time quantitative polymerase chain reaction, RT-qPCR）和蛋白质免疫印迹法（Western blot, WB）检测肿瘤组织分化抗原簇47（cluster of differentiation 47, CD47）信使核糖核酸（messenger ribonucleic acid, mRNA）和蛋白表达及巨噬细胞中信号调节蛋白α（signal regulatory protein α, SIRPα）表达。

**结果:**

三叶青多糖3个剂量组和脾多肽组抑瘤率和抗转移率高于模型组，肿瘤组织病理损伤严重，巨噬细胞吞噬墨汁阳性率和吞噬肿瘤细胞效率升高，巨噬细胞凋亡率下降，巨噬细胞增殖活性、巨噬细胞向M1型极化比例、DC抗原提呈能力、CD4^+^、CD4^+^/CD8^+^升高，血清肿瘤坏死因子α（tumor necrosis factor α, TNF-α）、白介素-1β（interleukin-1β, IL-1β）、肿瘤组织CD47、巨噬细胞蛋白酪氨酸磷酸酶1（SH2-containing protein tyrosine phosphatase 1, SHP-1）、蛋白酪氨酸磷酸酶2（SH2-containing protein tyrosine phosphatase 2, SHP-2）及磷酸化信号调节蛋白α（phosphorylation signal regulatory protein α, p-SIRPα）表达下降，差异均有统计学意义（P<0.05）。上述指标三叶青多糖低剂量组与脾多肽组的差异均无统计学意义（P>0.05），且三叶青多糖的作用均呈剂量依赖性。

**结论:**

三叶青多糖有抗Lewis肺癌小鼠肿瘤生长和转移及免疫反应的作用，其机制可能与抑制SIRP/CD47信号通路有关。

## Abstract (en)

**Background and objective:**

Lung cancer has a high incidence and mortality rate, but the treatment of lung cancer still lacks low toxicity and efficient anti-tumor drugs. Polysaccharide from radix tetrastigme has development value in anti-tumor treatment methods. This study was to observe the effect of polysaccharide from radix tetrastigme on immune response of Lewis lung cancer mice and explore its molecular mechanism.

**Methods:**

Lewis lung cancer mouse models were established and randomly grouped. The spleen polypeptide group was intragastric with 50 mg/kg spleen polypeptide, and the radix tetrastigme polysaccharide low, medium and high dose groups were intragastric with 62.5, 125 and 250 mg/kg radix tetrastigme polysaccharide, respectively, and the model group and the control group were intragastric with equivolume normal saline. Tumor formation and metastasis were compared. Haematoxylin-eosin (HE) staining was used to observe the pathological changes of tumor cells. Macrophage phagocytosis, apoptosis, M1/M2 polarization, T cell subsets and cytokine levels in peripheral blood were detected by flow cytometry. The proliferation activity of macrophages was detected by methyl thiazolyldiphenyl tetrazolium (MTT) assay. Dendritic cell (DC) antigen presenting function was detected by chlorophenol red-β-D-galactopyranoside (CPRG) method. Tumor tissue differentiation antigen cluster 47 (CD47) mRNA and protein expression and macrophage signal regulatory protein α (SIRRP α) expression were detected by real time quantitative polymerase chain reaction (RT-qPCR) and Western blot (WB).

**Results:**

The tumor inhibition rates and anti-metastasis rates in the 3-dose radix tetrastigme polysaccharide group and the spleen polypeptide group were higher than those in the model group, and the pathological injury of tumor tissue were severer, and the positive rate of phagocytosis of ink by macrophages and the efficiency of phagocytosis of tumor cells were increased; the apoptosis rate of macrophages was decreased; the proliferation activity of macrophages, polarization ratio of macrophages to M1 type, DC antigen presenting ability, CD4^+^, CD4^+^/CD8^+^ levels were increased; the level of serum tumor necrosis factor α (TNF-α), interleukin-1β (IL-1β), and the expression of tumor tissue CD47, macrophage SH2-containing protein tyrosine phosphatase 1 (SHP-1), SH2-containing protein tyrosine phosphatase 2 (SHP-2), and phosphorylation signal regulatory protein α (p-SIRPα) were decreased, and the differences were statistically significant (P<0.05). There were no significant differences in the above indexes between low-dose radix tetrastigme polysaccharide group and spleen polypeptide group (P>0.05), and the effects of radix tetrastigme polysaccharide were dose-dependent.

**Conclusion:**

Radix tetrastigme polysaccharide can inhibit tumor growth, metastasis and immune response in Lewis lung cancer mice, and its mechanism may be related to inhibiting SIRP/CD47 signaling pathway.

近几十年来肺癌是全球范围最常见的癌症，因其高致病率和高致死率，严重威胁人类生命^[1]^。当前癌症治疗方式中手术和放射治疗周期短且疗效明确，但长期治疗易导致机体免疫功能受损，进一步导致癌细胞转移，且药物治疗虽具有一定疗效，但因其耐药性和毒副作用迫使研究者寻找低毒高效的抗肿瘤药物^[2]^。三叶青是我国特有的珍稀中药药材，具有清热、抗炎和抗病毒等功效^[3]^。目前，有研究^[4⇓-6]^报道三叶青多糖可抑制肝癌细胞增殖，诱导人肝癌细胞凋亡，对乳腺癌小鼠有抗肿瘤作用，还可抑制非小细胞肺癌细胞增殖。由于肿瘤研究和免疫研究的深入和交叉渗透，肿瘤免疫治疗迅猛发展。肿瘤免疫治疗激活机体免疫系统，靶向控制和杀灭肿瘤细胞，为肿瘤患者提供具有更高疗效性和安全性的治疗方式。免疫系统可通过免疫细胞清除机体肿瘤细胞^[7,8]^；免疫检查点是机体的免疫负调节分子，可避免机体异常免疫应答造成的自身组织损伤^[9]^，而免疫检查点抑制剂可有效治疗肿瘤^[10]^。分化抗原簇47（cluster of differentiation 47, CD47）又称整合素相关蛋白，是免疫球蛋白超家族的一员，在正常细胞和肿瘤细胞中均有表达，也是机体的免疫检查点之一，作为免疫检查点受体的信号调节蛋白α（signal regulatory protein α, SIRPα）的胞外N端可与CD47结合介导双向信号传导，诱导免疫受体酪氨酸抑制性基序（immunoreceptor tyrosine-based inhibition motifs, ITIM）磷酸化，在调控机体免疫应答及肿瘤的发生发展过程中发挥重要作用^[10]^。SIRPα作为免疫球蛋白超家族的跨膜蛋白，是CD47的重要表面受体，正常生理条件下CD47与巨噬细胞表面SIRPα结合，向巨噬细胞传递抑制性信号，使其不会错误吞噬正常细胞^[11]^。在恶性肿瘤细胞中CD47过表达，可与巨噬细胞表面的SIRPα相结合，从而避免巨噬细胞对其产生免疫排斥，促使恶性肿瘤细胞免疫逃逸。研究^[12]^证明在肺癌发生发展中CD47/SIRPα信号通路被激活，而抑制该通路有助于增强巨噬细胞吞噬癌细胞的能力，达到抗肿瘤作用。既往研究^[13]^显示，柴胡多糖可降低CD47、SIRPα的表达，从而增强巨噬细胞的杀伤作用。三叶青多糖和柴胡多糖同属于从中药中提取的多糖，故推测三叶青多糖对CD47/SIRPα信号通路具有调控作用。然而三叶青多糖能否通过该信号通路调控肺癌抗肿瘤免疫反应尚未可知。鉴于此，本研究取C57BL/6J小鼠开展动物实验，为三叶青多糖治疗肺癌提供理论基础。

## 1 材料与方法

### 1.1 实验材料

#### 1.1.1 实验动物与瘤株

C57BL/6J小鼠60只，无特定病原体（specific pathogen free, SPF）级，6-8周龄，雌雄各半，体重为（20.0±2.0）g，购自陆军军医大学基础医学院实验动物学教研室，实验动物生产许可证号：SCXK（渝）2022-0011。Lewis肺癌细胞由上海素尔生物科技有限公司引进，液氮冻存。本研究经重庆市长寿区人民医院动物伦理委员会审批（审批号：院准字2022年第003号）。

#### 1.1.2 实验药材和试剂

脾多肽（购自西安辰辉康泽生物科技有限公司，生产批号：CH2141）；三叶青多糖（购自西安晋恒化工有限公司，生产批号：JH01，提纯后经高效液相色谱法鉴定纯度≥98%^[14]^）；苏木素-伊红（haematoxylin-eosin, HE）染色试剂盒（购自索莱宝，生产批号：G1120）；金黄色葡萄球菌标准菌株（购自上海复祥生物科技有限公司，生产批号：ATCC25909）；墨汁（购自泾县周记宣纸工艺厂，生产批号：176）；大鼠抗小鼠聚乙烯（polyethylene, PE）标记F4/80（F4/80-PE）抗体（购自艾美捷科技有限公司，生产批号：HM1066PE-100）；大鼠抗小鼠异硫氰酸荧光素（fluorescein isothiocyanate, FITC）标记CD16/32（CD16/32-FITC）抗体、CD206-FITC抗体（购自武汉伊莱瑞特生物科技股份有限公司，生产批号：E-AB-F0997UC、E-AB-F1135C）；CD47基因和内参β-肌动蛋白（β-actin）基因所需实时荧光定量逆转录聚合酶链反应（real time quantitative polymerase chain reaction, RT-qPCR）引物（由上海卓强生物科技有限公司合成）；β-肌动蛋白（β-actin）单克隆抗体（购自亚科因武汉生物技术有限公司，生产批号：A01010）；预染蛋白标记（Marker）（购自美国赛默飞世尔公司，生产批号：26619）。

#### 1.1.3 实验设备

R201旋转式蒸发仪（购自上海申顺生物科技有限公司）；FACSAria II型流式细胞仪（购自美国BD）；7500型实时荧光定量PCR仪（购自北京赛百奥科技有限公司）；JS25-SH-2040型凝胶电泳系统（购自北京中西华大科技有限公司）；Image-Pro Plus v6.0软件（购自美国Media Cybernetics公司）。

### 1.2 方法

#### 1.2.1 构建Lewis肺癌小鼠模型

将冻存于液氮中的Lewis肺癌细胞复苏传代，调整细胞密度为1×10^7^个/mL，将Lewis肺癌细胞悬液接种于50只C57BL/6J小鼠右腋皮下，每只0.1 mL，构建Lewis肺癌小鼠模型。1周后观察建模组小鼠，通过肉眼观察其皮下移植瘤呈囊实性，且通过游标卡尺测得移植瘤体积>1 mm×1 mm×1 mm表明Lewis肺癌小鼠模型构建成功^[15]^。剩余10只正常健康C57BL/6J小鼠于右腋皮下注射0.1 mL生理盐水，标记为对照组。

#### 1.2.2 小鼠分组及药物干预

依据随机数字表法将建模成功的小鼠分为脾多肽组、三叶青多糖低、中、高剂量组和模型组。建模24 h后灌胃给药，对照组和模型组分别予以0.1 mL/kg生理盐水，脾多肽组予以50 mg/kg脾多肽溶于等容积生理盐水中灌胃^[16]^，三叶青多糖低、中、高剂量组分别予以62.5、125、250 mg/kg三叶青多糖溶于等容积生理盐水中灌胃^[5]^，每天给药1次，持续3周。

#### 1.2.3 观察各组肿瘤体积并计算抑瘤率

停药24 h后，断颈处死小鼠，游标卡尺测量肿瘤组织长径及垂直径，肿瘤体积=1/2长径×短径^2^，抑瘤率=（模型组瘤重-治疗组瘤重）/模型组瘤重×100.00%。

#### 1.2.4 HE染色观察肿瘤细胞形态学变化及肺组织肿瘤灶

取肿瘤组织，固定于10%甲醛溶液，常规脱色、包埋、切片（5 μm），行HE染色：脱蜡至水，苏木素染色10 min，分化，伊红复染1 min，脱水脱透明封片，光学显微镜观察肿瘤细胞形态学变化；同步骤取小鼠肺组织行HE染色，观察肺组织中肿瘤灶结节，并计算抗转移率。抗转移率=（模型组平均肺转移灶数-治疗组平均肺转移灶数）/模型组平均肺转移灶数×100.00%^[17]^。

#### 1.2.5 墨汁吞噬试验及巨噬细胞对肿瘤细胞的吞噬作用、凋亡水平、增殖活性

##### 1.2.5.1 巨噬细胞分离培养、制备悬液

给药结束24 h后，75%乙醇浸泡断颈处死的小鼠，向小鼠腹腔注入无血清RPMI-1640，5 min后从小鼠腹腔两侧抽取腹腔液，1000 g 4 ℃离心10 min，加10 mL Eagle培养基，洗涤，获得巨噬细胞。倒置显微镜下计数，调整至2.5×10^6^/mL细胞浓度获得巨噬细胞悬液。将巨噬细胞悬液置于6孔板和24孔板内，37 ^o^C培养箱中培养，12 h后保留贴壁细胞，去除少量未贴壁杂细胞，培养用以观察巨噬细胞对肿瘤细胞的吞噬作用、巨噬细胞的增殖活性和极化水平。

##### 1.2.5.2 墨汁吞噬试验

各组巨噬细胞培养48 h后，每孔加入0.02 mL稀释2倍的墨汁，6孔板继续37 ^o^C培养箱中培养约4 h。当出现墨汁吞入现象时，无血清细胞培养液洗涤细胞，10%甲醛固定，甘油明胶封片，加摄像头的倒置显微镜观察并拍照。Image-Pro Plus v6.0图像分析软件计算吞噬墨汁阳性率^[18]^。

##### 1.2.5.3 流式细胞术检测巨噬细胞对肿瘤细胞的吞噬作用、凋亡水平

取Lewis肺癌细胞，调整细胞密度为1×10^7^个/mL，加入羟基荧光素二醋酸盐琥珀酰亚胺脂（5,6- carboxyfluorescein diacetate, succinimidyl ester, CFSE），37 ^o^C孵育20 min后离心收集细胞；各组巨噬细胞培养48 h后与CFSE标记的Lewis肺癌细胞以3:1比例混合，在37 ^o^C、5% CO_2_条件下共培养6 h；采用F4/80-藻青蛋白（allophycocyanin, APC）进行染色，流式细胞仪检测巨噬细胞吞噬肿瘤细胞的吞噬效率，吞噬效率=F4/80^+^CFSE^+^细胞/F4/80^+^细胞×100.00%。

各组巨噬细胞培养48 h后，接种至6孔板，培养过夜，离心取沉淀，结合缓冲液重悬细胞后各加5 μL Annexin V-FITC和碘化丙啶（propidium iodide, PI）溶液，室温避光孵育15 min，上机测细胞凋亡率。将第二象限和第四象限细胞占比记为凋亡率。

##### 1.2.5.4 噻唑蓝溴化四唑（methyl thiazolyldiphenyl tetrazolium, MTT）法检测巨噬细胞增殖活性

各组巨噬细胞在Eagle培养基中培养48 h后，调整密度为1×10^8^个/mL，然后接种至96孔板，每孔100 μL，室温培养24 h，加10 μL MTT溶液孵育4 h，每孔加二甲基亚砜150 μL，孵育10 min后用酶标仪测490 nm处各组细胞吸光度值。

#### 1.2.6 流式细胞术鉴别巨噬细胞M1/M2型极化水平

取1.2.5所获取的巨噬细胞悬液，胰酶消化后调整每管密度5×10^5^个/流式管，加5 μL大鼠抗小鼠F4/80-PE抗体、5 μL大鼠抗小鼠CD16/32-FITC抗体、5 μL大鼠抗小鼠CD206-FITC抗体，4 ^o^C孵育30 min，洗去结合抗体，上流式细胞仪检测巨噬细胞M1/M2型极化水平。流式数据采用FlowJo进行分析。

#### 1.2.7 β-D-吡喃半乳糖苷氯酚红（chlorophenol red-β-D-galactopyranoside, CPRG）法检测树突状细胞（dendritic cell, DC）抗原提呈功能

取小鼠双侧胫骨与股骨，用无菌RPMI-1640培养基反复冲洗骨髓腔，将骨髓细胞悬液经200目细胞滤网过滤收集单细胞，加红细胞裂解液裂解红细胞，离心去上清，调整细胞密度为1×10^6^个/mL，接种至12孔板（1 mL/孔）；分别加终浓度为20 ng/mL粒细胞-巨噬细胞集落刺激因子（granulocyte-macrophage colony-stimulating factor, GM-CSF）、5 ng/mL白介素-4（interleukin-4, IL-4），10%胎牛血清，100 U/mL青-链霉素，37 ^o^C、5% CO_2_条件培养；铺板当天记为第1天，第3、5天分别半量换液，第6天获得不成熟DC，加完全培养基、10 ng/mL肿瘤坏死因子α（tumor necrosis factor α, TNF-α）、1 μg/mL聚乙二醇（polyethylene glycol 2, PEG2）、10 ng/mL白介素-6（interleukin-6, IL-6）、10 ng/mL白介素-1β（interleukin-1β, IL-1β）重悬细胞，培养24 h后获得成熟DC。取各组成熟DC细胞，加0.1 mL细胞密度为2×10^6^个/mL的B3Z细胞，孵育过夜；加CPRG工作液，常温孵育4 h后加终止液，离心取上清，酶标仪检测595 nm处吸光度值。

#### 1.2.8 流式细胞术检测外周血T细胞亚群水平

取小鼠腹主动脉血1 mL，加入CD4 PE和CD8 FITC标记抗体，避光孵育30 min后，重悬细胞，上机检测小鼠外周血T细胞亚群水平。

#### 1.2.9 酶联免疫吸附试验（enzyme linked immunosorbent assay, ELISA）法检测外周血TNF-α、IL-1β水平

取小鼠腹主动脉血1 mL，参照试剂盒操作说明检测小鼠血清TNF-α、IL-1β水平。

#### 1.2.10 RT-qPCR检测肿瘤组织CD47信使核糖核酸（messenger ribonucleic acid, mRNA）表达水平

提取对照组肺组织和建模组肿瘤组织总RNA。逆转录后进行实时荧光定量PCR。β-actin作为内参，检测肺肿瘤组织中CD47 mRNA相对表达水平。引物序列：CD47，正义链5’-CTTTGAAGAGATGAGCAGTG-3’，反义链5’-CGATGTGGGATCTAATTCTC-3’；β-actin，正义链5’-TGGATCAGCAAGCAGGAGTA-3’，反义链5’- TCGGCCACATTGTGAACTTT-3’。反应条件为：96 ^o^C预变性20 s；94 ^o^C 30 s、55 ^o^C 20 s、72 ^o^C 20 s，45个循环；95 ^o^C 1 min、60 ^o^C 30 s、95 ^o^C 30 s。根据相对定量法^[19]^比较各组CD47 mRNA表达差异。以Folds=2^-ΔΔCt^表示各组与对照组中CD47 mRNA表达的倍比关系。

#### 1.2.11 蛋白质免疫印迹法（Western blot, WB）检测肿瘤组织CD47蛋白、巨噬细胞表面SIRPα、蛋白酪氨酸磷酸酶（SH2-containing protein tyrosine phosphatase, SHP）的SHP-1、SHP-2及磷酸化信号调节蛋白α（phosphorylation signal regulatory protein α, p-SIRPα）水平检测

获取对照组正常肺组织，其余各组肺肿瘤组织蛋白。获取少量相应组织块，细胞裂解液和超声仪裂解，上清即为组织全蛋白。倒掉巨噬细胞培养液，PBS洗涤，细胞裂解液冰上裂解，离心，上清即为细胞全蛋白。等量蛋白质进行凝胶电泳，转膜后迅速加入脱脂奶粉，室温封闭1 h。将聚偏二氟乙烯（polyvinylidene fluoride, PVDF）膜放入CD47（1:1000）、SIRPα（1:1000）、p-SIRPα（1:1000）、SHP-1（1:1000）、SHP-2（1:500）一抗中，摇床4^ o^C过夜。次日，将PVDF膜放入含有羊抗兔二抗（1:5000）的离心管中，室温下用摇床孵育，最终进行化学发光反应。利用超高灵敏度化学发光成像系统曝光。使用Image J pro Plus 5.0软件检测蛋白印迹灰度值。

### 1.3 统计学分析

SPSS 22.0软件用于统计学分析。实验数据经Shapiro-Wilk检验均符合正态分布，以均数±标准差表示。多组间差异比较采用单因素方差分析和SNK-q检验。P<0.05为差异有统计学意义。

## 2 结果

### 2.1 各组小鼠肿瘤体积、瘤重和抑瘤率比较

观察建模小鼠，显示皮下移植瘤呈囊实性，提示建模成功。建模小鼠成瘤率为100.00%。将剩余49只建模小鼠采用随机数字表法分为模型组9只及脾多肽组、三叶青多糖低、中和高剂量组各10只。与模型组比较，脾多肽组和三叶青多糖3个剂量组肿瘤体积和瘤重显著减小、抑瘤率显著增高（P<0.01）。脾多肽组与三叶青多糖低剂量组肿瘤体积、瘤重和抑瘤率差异无统计学意义（P>0.05）。三叶青多糖肿瘤体积和抑瘤率呈剂量依赖性。三叶青多糖低、中、高剂量组间差异有统计学意义（P<0.05）。见图1、表1。

**图1:**
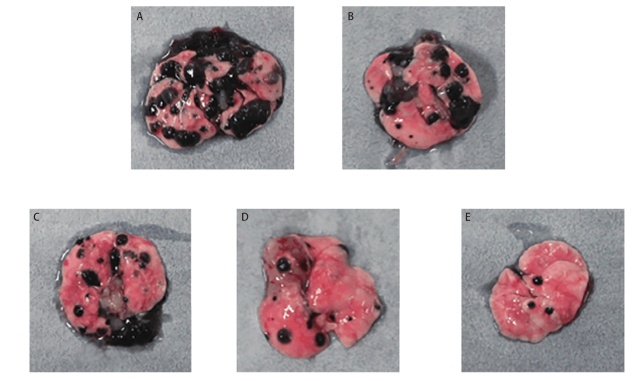
各组小鼠皮下移植瘤瘤体图。A：模型组；B：脾多肽组；C：三叶青多糖低剂量组；D：三叶青多糖中剂量组；E：三叶青多糖高剂量组。

**表1:** 各组小鼠肿瘤体积、瘤重、抑瘤率比较

Groups	n	Tumor volume (cm^3^)	Tumor weight (%)	Tumor inhibitation rate (%)
Model group	9	0.52±0.05	14.12±2.38	-
Spleen polypeptide group	10	0.47±0.06^##^	13.25±2.01^##^	6.16±1.73^##^
Radix tetrastigme polysaccharide low dose group	10	0.45±0.07^##^	13.23±1.99^##^	6.30±1.70^##^
Radix tetrastigme polysaccharide medium dose group	10	0.34±0.05^##△△^	9.31±1.78^##△△^	34.07±5.86^##△△^
Radix tetrastigme polysaccharide high dose group	10	0.28±0.04^##△△^	7.60±1.50^##△△^	46.18±7.69^##△△^
*F*		24.065	26.378	20.182
*P*		<0.001	<0.001	<0.001

^##^P<0.01 vs model group; ^△△^P<0.01 vs spleen polypeptide group.

### 2.2 HE染色观察肿瘤细胞形态学变化及肺组织中肿瘤灶情况

模型组小鼠肿瘤细胞排列紧密、肿瘤细胞形态正常；脾多肽组和三叶青多糖低剂量组肿瘤细胞排列变疏松、不规则、细胞破裂坏死；三叶青多糖中剂量组肿瘤细胞排列进一步疏松、细胞坏死数量增加；三叶青多糖高剂量组肿瘤细胞坏死数量最多（图2）。模型组、脾多肽组及三叶青多糖低、中、高剂量组出现肺转移的小鼠分别有8、8、8、5、4只。与模型组比较，脾多肽组和三叶青多糖3个剂量组肿瘤抗转移率显著增高（P<0.01），脾多肽组与三叶青多糖低剂量组抗转移率差异无统计学意义（P>0.05）。三叶青多糖抗转移率呈剂量依赖性。三叶青多糖低、中、高剂量组间差异有统计学意义（P<0.05）。见表2。

**图2:**
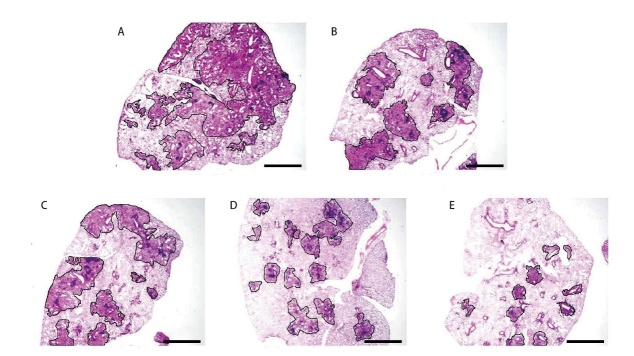
各组小鼠肿瘤组织细胞形态学变化（HE染色，比例尺1 mm）。A：模型组；B：脾多肽组；C：三叶青多糖低剂量组；D：三叶青多糖中剂量组；E：三叶青多糖高剂量组。

**表2:** 各组小鼠肿瘤抗转移率比较

Groups	n	Anti-metastasis rate (%)
Model group	9	-
Spleen polypeptide group	10	47.29±1.86^##^
Radix tetrastigme polysaccharide low dose group	10	48.36±1.76^##^
Radix tetrastigme polysaccharide medium dose group	10	62.59±1.93^##△△^
Radix tetrastigme polysaccharide high dose group	10	46.37±1.47^##△△^
*F*		37.806
*P*		<0.001

^##^P<0.01 vs model group; ^△△^P<0.01 vs spleen polypeptide group.

### 2.3 各组小鼠巨噬细胞吞噬能力、凋亡水平及增殖活性比较

与对照组相比，实验组（包括模型组、脾多肽组及三叶青多糖3个剂量组）巨噬细胞吞噬墨汁颗粒数量和肿瘤细胞效率、增殖活性显著降低，凋亡率显著升高（P<0.05）。三叶青多糖3个剂量组和脾多肽组吞噬墨汁颗粒数和肿瘤细胞效率、增殖活性较模型组显著增多，凋亡率较模型组显著降低（P<0.01）。三叶青多糖低剂量组与脾多肽组的巨噬细胞吞噬墨汁颗粒数量和肿瘤细胞效率、增殖活性、凋亡率差异无统计学意义（P>0.05）。三叶青多糖上调吞噬墨汁颗粒数和肿瘤细胞效率、增殖活性及下调凋亡率呈剂量依赖性。三叶青多糖低、中、高剂量组间差异有统计学意义（P<0.05）。见图3、图4和表3。

**图3:**
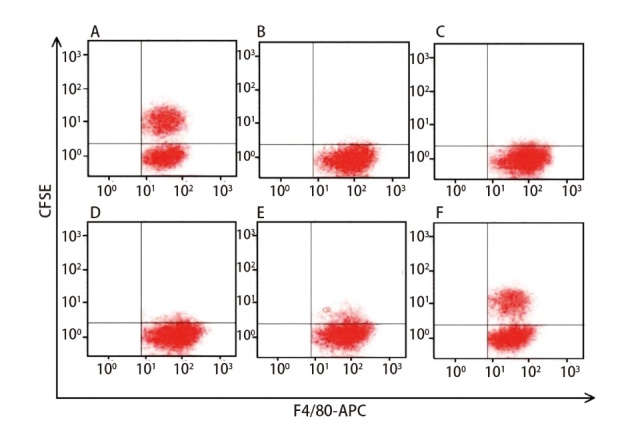
流式细胞术检测巨噬细胞吞噬肿瘤细胞效率。A：对照组；B：模型组；C：脾多肽组；D：三叶青多糖低剂量组；E：三叶青多糖中剂量组；F：三叶青多糖高剂量组。

**图4:**
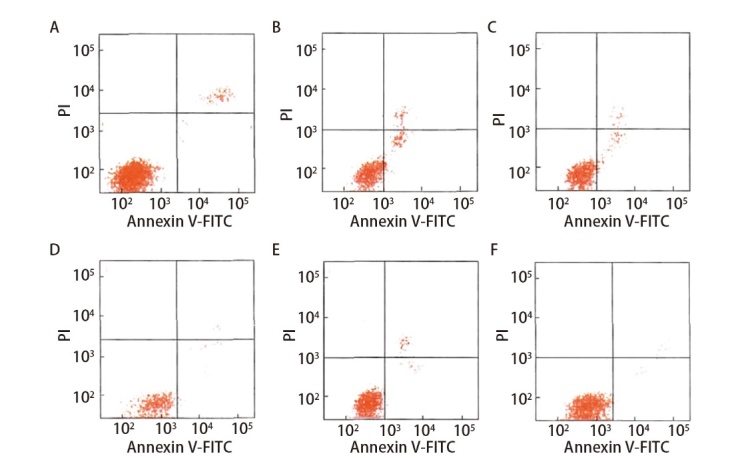
流式细胞术检测巨噬细胞凋亡率。A：对照组；B：模型组；C：脾多肽组；D：三叶青多糖低剂量组；E：三叶青多糖中剂量组；F：三叶青多糖高剂量组。

**表3:** 各组小鼠吞噬细胞中吞噬墨汁、肿瘤细胞效率测定结果、巨噬细胞凋亡率和增殖活性比较

Groups	Phagocytic ink positive rates (%)	Phagocytosis efficiency (%)	Acrophage apoptosis rate (%)	Macrophage proliferation activity
Control group	4.39±0.58	42.85±7.32	6.07±1.15	0.62±0.15
Model group	1.02±0.14^**^	4.69±0.85^**^	33.19±5.17^**^	0.30±0.04^**^
Spleen polypeptide group	1.69±0.28^**##^	7.15±1.72^**##^	24.62±4.81^**##^	0.39±0.05^**##^
Radix tetrastigme polysaccharide low dose group	1.68±0.27^**##^	8.63±2.07^**##^	25.58±4.97^**##^	0.40±0.05^**##^
Radix tetrastigme polysaccharide medium dose group	2.98±0.53^**##△△^	16.59±2.35^**##△△^	14.49±2.35^**##△△^	0.46±0.07^**##△△^
Radix tetrastigme polysaccharide high dose group	3.52±0.87^*##△△^	36.17±5.89^**##△△^	6.91±1.68^**##△△^	0.55±0.08^**##△△^
*F*	15.193	22.765	18.106	24.633
*P*	<0.001	<0.001	<0.001	<0.001

^*^P<0.05, ^**^P<0.01 vs control group; ^##^P<0.01 vs model group; ^△△^P<0.01 vs spleen polypeptide group.

### 2.4 各组小鼠巨噬细胞M1/M2极化水平比较

与对照组相比，实验组（包括模型组、脾多肽组及三叶青多糖3个剂量组）F4/80-PE/CD16/32-FITC双阳性细胞（M1型）比例显著减少（P<0.05），F4/80-PE/CD206-FITC双阳性细胞（M2型）比例显著增加（P<0.01）；与模型组比较，脾多肽和三叶青多糖3个剂量组M1型巨噬细胞比例显著增加（P<0.01），M2型巨噬细胞比例显著减少（P<0.01）。三叶青多糖增加M1型巨噬细胞比例、减少M2型巨噬细胞比例呈剂量依赖性。三叶青多糖低、中、高剂量组间差异有统计学意义（P<0.01）。见图5、表4。

**图5:**
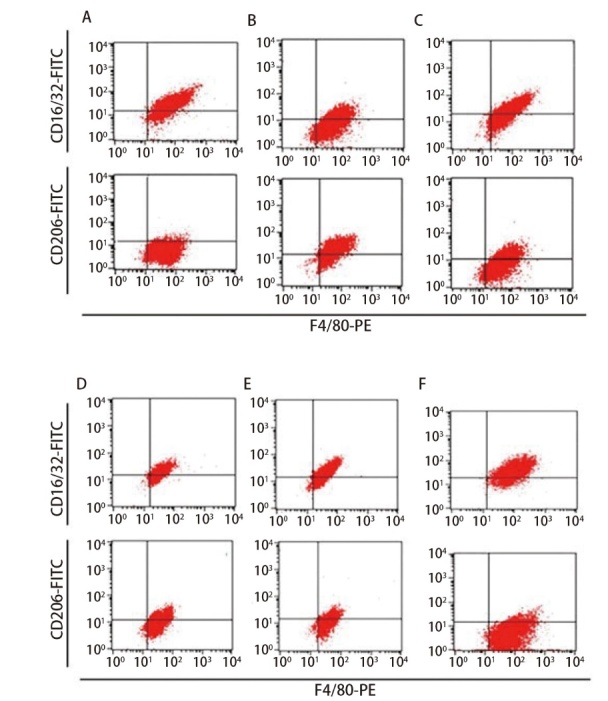
流式细胞仪检测小鼠巨噬细胞M1/M2极化水平。A：对照组；B：模型组；C：脾多肽组；D：三叶青多糖低剂量组；E：三叶青多糖中剂量组；F：三叶青多糖高剂量组。

**表4:** 各组小鼠巨噬细胞中M1/M2极化水平、DC抗原提呈能力比较

Groups	M1 (%)	M2 (%)	Absorbance values
Control group	79.92±9.20	1.67±0.31	0.68±0.12
Model group	31.25±3.48^**^	46.83±7.16^**^	0.22±0.04^**^
Spleen polypeptide group	47.09±6.88^**##^	30.91±5.38^**##^	0.30±0.05^**##^
Radix tetrastigme polysaccharide low dose group	48.65±6.73^**##^	29.74±5.74^**##^	0.29±0.06^**##^
Radix tetrastigme polysaccharide medium dose group	61.82±7.16^**##△△^	13.72±3.12^**##△△^	0.41±0.07^**##△△^
Radix tetrastigme polysaccharide high dose group	77.59±8.35^*##△△^	5.73±1.17^**##△△^	0.57±0.08^**##△△^
*F*	18.619	21.257	19.382
*P*	<0.001	<0.001	<0.001

^*^P<0.05, ^**^P<0.01 vs control group; ^##^P<0.01 vs model group; ^△△^P<0.01 vs spleen polypeptide group. DC: dentritic cell.

### 2.5 各组小鼠DC抗原提呈能力检测

与对照组相比，实验组（包括模型组、脾多肽组及三叶青多糖3个剂量组）DC抗原提呈能力显著下降（P<0.01）；与模型组比较，脾多肽和三叶青多糖3个剂量组DC抗原提呈能力增强（P<0.05）。三叶青多糖增强DC抗原提呈能力呈剂量依赖性。三叶青多糖低、中、高剂量组间差异有统计学意义（P<0.01）。见表4。

### 2.6 各组小鼠外周血T细胞亚群

与对照组相比，实验组（包括模型组、脾多肽组及三叶青多糖3个剂量组）CD4^+^、CD4^+^/CD8^+^水平显著下降（P<0.05），CD8^+^水平显著升高（P<0.01）；与模型组比较，脾多肽和三叶青多糖3个剂量组CD4^+^、CD4^+^/CD8^+^水平升高，CD8^+^水平下降（P<0.05）。三叶青多糖上调CD4^+^、CD4^+^/CD8^+^水平及下调CD8^+^水平呈剂量依赖性。三叶青多糖低、中、高剂量组间差异有统计学意义（P<0.05）。见图6、表5。

**图6:**
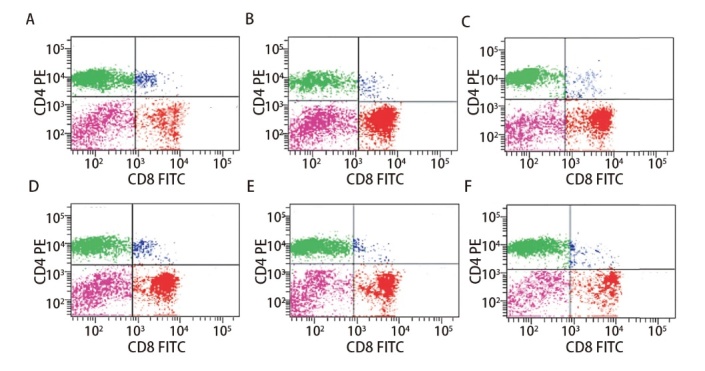
流式细胞术检测外周血T细胞亚群水平。A：对照组；B：模型组；C：脾多肽组；D：三叶青多糖低剂量组；E：三叶青多糖中剂量组；F：三叶青多糖高剂量组。

**表5:** 各组小鼠T细胞亚群水平比较

Groups	CD4^+ ^(%)	CD8^+^ (%)	CD4^+^/CD8^+^
Control group	56.65±6.78	27.27±3.16	2.08±0.34
Model group	28.77±3.56^**^	42.06±5.19^**^	0.68±0.11^**^
Spleen polypeptide group	35.64±4.62^**##^	37.40±4.76^**##^	0.95±0.15^**##^
Radix tetrastigme polysaccharide low dose group	36.43±4.55^**##^	37.01±4.58^**##^	0.98±0.16^**##^
Radix tetrastigme polysaccharide medium dose group	43.92±4.59^**##△△^	32.27±3.24^**##△△^	1.36±0.22^**##△△^
Radix tetrastigme polysaccharide high dose group	49.73±5.17^*##△△^	28.37±3.17^**##△△^	1.75±0.30^**##△△^
*F*	24.675	22.105	19.376
*P*	<0.001	<0.001	<0.001

^*^P<0.05, ^**^P<0.01 vs control group; ^##^P<0.01 vs model group; ^△△^P<0.01 vs spleen polypeptide group.

### 2.7 各组小鼠外周血TNF-α、IL-1β水平

与对照组相比，实验组（包括模型组、脾多肽组及三叶青多糖3个剂量组）血清TNF-α、IL-1β水平显著升高（P<0.05）；与模型组比较，脾多肽和三叶青多糖3个剂量组TNF-α、IL-1β水平下降（P<0.05）。三叶青多糖下调TNF-α、IL-1β水平呈剂量依赖性。三叶青多糖低、中、高剂量组间差异有统计学意义（P<0.05）。见表6。

**表6:** 各组小鼠血清TNF-α、IL-1β水平比较

Groups	TNF-α (pg/mL)	IL-6 (pg/mL)
Control group	22.11±4.10	30.15±5.83
Model group	92.35±11.88^**^	98.73±12.95^**^
Spleen polypeptide group	72.06±10.30^**##^	74.69±10.38^**##^
Radix tetrastigme polysaccharide low dose group	73.74±10.19^**##^	73.12±9.82^**##^
Radix tetrastigme polysaccharide medium dose group	50.02±8.10^**##△△^	51.06±7.86^**##△△^
Radix tetrastigme polysaccharide high dose group	44.67±7.02^*##△△^	45.24±7.56^**##△△^
*F*	14.203	12.558
*P*	<0.001	<0.001

^*^P<0.05, ^**^P<0.01 vs control group; ^##^P<0.01 vs model group; ^△△^P<0.01 vs spleen polypeptide group; TNF-α: tumor necrosis factor α; IL-1β: interleukin-1β.

### 2.8 各组小鼠肿瘤组织CD47 mRNA和蛋白表达量比较

与对照组相比，模型组CD47 mRNA表达显著升高，脾多肽组及三叶青多糖3个剂量组CD47 mRNA表达显著下降，实验组（包括模型组、脾多肽组及三叶青多糖3个剂量组）CD47蛋白表达显著升高（P<0.05）；与模型组比较，脾多肽和三叶青多糖3个剂量组CD47 mRNA和蛋白表达显著下降（P<0.01）。脾多肽组与三叶青多糖低剂量组CD47表达差异无统计学意义（P>0.05）。三叶青多糖降低CD47表达呈剂量依赖性。三叶青多糖低、中、高剂量组间差异有统计学意义（P<0.05）。见图7、表7。

**图7:**
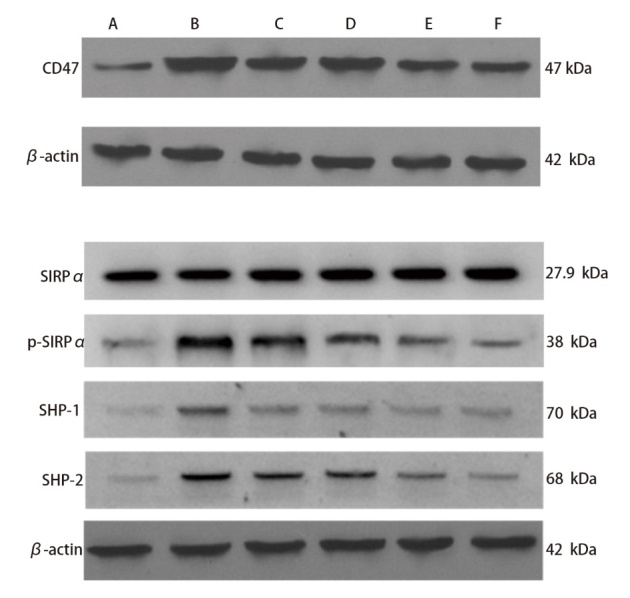
Western blot检测各组小鼠CD47蛋白和SIRPα蛋白的相对表达量。A：对照组；B：模型组；C：脾多肽组；D：三叶青多糖低剂量组；E：三叶青多糖中剂量组；F：三叶青多糖高剂量组。

**表7:** 各组小鼠CD47表达量和SIRPα、SHP-1、SHP-2蛋白相对表达量及p-SIRPα水平比较

Groups	CD47 mRNA	CD47 protein	SIRPα	SHP-1	SHP-2	p-SIRPα
Control group	1.00	0.49±0.05	0.98±0.16	0.12±0.03	0.09±0.03	0.15±0.03
Model group	1.25±0.14^**^	1.56±0.31^**^	0.96±0.18	0.79±0.14^**^	0.75±0.12^**^	0.86±0.15^**^
Spleen polypeptide group	0.61±0.11^**##^	1.20±0.30^**##^	0.97±0.17	0.45±0.05^**##^	0.43±0.05^**##^	0.50±0.07^**##^
Radix tetrastigme polysaccharide low dose group	0.60±0.10^**##^	1.22±0.32^**##^	0.98±0.16	0.47±0.06^**##^	0.44±0.07^**##^	0.49±0.08^**##^
Radix tetrastigme polysaccharide medium dose group	0.52±0.07^**##△△^	0.96±0.17^**##△△^	0.98±0.17	0.35±0.05^**##△△^	0.31±0.06^**##△△^	0.41±0.07^**##△△^
Radix tetrastigme polysaccharide high dose group	0.51±0.07^*##△△^	0.78±0.15^**##△△^	0.96±0.17	0.28±0.04^**##△△^	0.25±0.05^**##△△^	0.34±0.05^**##△△^
*F*	15.339	18.675	1.125	14.693	18.524	17.692
*P*	<0.001	<0.001	0.278	<0.001	<0.001	<0.001

^*^P<0.05, ^**^P<0.01 vs control group; ^##^P<0.01 vs model group; ^△△^P<0.01 vs spleen polypeptide group.

### 2.9 各组小鼠巨噬细胞SIRPα、SHP-1、SHP-2蛋白表达量及p-SIRPα水平比较

与对照组比较，实验组（包括模型组、脾多肽组及三叶青多糖3个剂量组）SHP-1、SHP-2表达量及p-SIRPα水平显著升高（P<0.01）；与模型组比较，脾多肽和三叶青多糖3个剂量组SHP-1、SHP-2表达量及p-SIRPα水平显著下降（P<0.01）。脾多肽组与三叶青多糖低剂量组SHP-1、SHP-2蛋白表达及p-SIRPα水平差异无统计学意义（P>0.05）。三叶青多糖升高SHP-1、SHP-2蛋白表达及p-SIRPα水平呈剂量依赖性。三叶青多糖低、中、高剂量组间差异有统计学意义（P<0.05）。见图7、表7。

## 3 讨论

脾多肽是一种免疫增强药，能够提高机体免疫力，在临床上可用于治疗肺癌等各种原发性转移性恶性肿瘤^[20]^。有研究^[21]^指出脾多肽可调控巨噬细胞发挥功能。本研究将脾多肽作为研究阳性药物，与三叶青多糖给药处理形成对照实验，保证研究的完整性和可靠性。巨噬细胞能够吞噬细菌，与机体的非特异性免疫功能相关，研究^[22]^指出在肿瘤发展时巨噬细胞噬菌功能受到抑制；当巨噬细胞处于M2型极化状态时会抑制肿瘤免疫，促进肿瘤生长和转移，而当巨噬细胞处于M1型极化状态时促进巨噬细胞的吞噬能力，激发肿瘤免疫反应^[23]^。本研究结果与上述研究一致，提示三叶青多糖可能通过增强巨噬细胞吞噬异物能力，增强机体非特异性免疫功能，抑制肿瘤的发生和发展。

DC、巨噬细胞和T细胞参与恶性肿瘤的发生和发展过程，其中DC和巨噬细胞均属于抗原递呈细胞，当DC细胞活化，巨噬细胞向M2型极化漂移，可使得SIRPα磷酸化水平下降，增加与肿瘤细胞表达的CD47特异性结合水平，从而抑制肿瘤细胞生长和增殖，增强巨噬细胞对肿瘤细胞的识别能力及吞噬作用；与此同时，T细胞被激活，成为细胞毒性T细胞，对肿瘤细胞的杀伤效应增强。CD4^+^、CD8^+^属于T细胞亚群，CD4^+ ^T细胞分泌TNF-α等细胞因子，介导细胞免疫，起抗肿瘤作用；CD8^+ ^T细胞能够直接杀伤肿瘤细胞，正常情况下两种细胞维持动态平衡，保持机体正常的免疫功能。肿瘤患者免疫功能紊乱，CD4^+ ^T细胞水平下降，CD8^+ ^T细胞水平升高^[5]^。IL-1β由巨噬细胞分泌，在肿瘤细胞中表达增强^[24]^。本研究结果表明三叶青多糖可上调外周血CD4^+^、CD4^+^/CD8^+^水平，降低TNF-α、IL-1β水平，抑制肿瘤细胞CD47的表达，并且抑制SHP-1、SHP-2及巨噬细胞表面p-SIRPα的表达，提示三叶青多糖可能通过调节免疫细胞及细胞因子的分泌增强DC、巨噬细胞和T细胞对肿瘤细胞的杀伤活性，从而达到抑制肿瘤生长的目的。

在生理条件下，正常细胞表达的CD47与巨噬细胞表面SIRPα结合，保证正常细胞不被巨噬细胞吞噬，而在肿瘤细胞中CD47表达显著上升，过量表达的CD47结合巨噬细胞上的SIRPα，促使SIRPα被磷酸化招募SHP-1/2，促使癌细胞免疫逃逸，造成机体损伤^[11,25]^。有研究^[26]^指出阻断CD47/SIRPα相互作用，抑制巨噬细胞表面SIRPα磷酸化水平，可增强巨噬细胞吞噬肿瘤细胞的能力，抑制肿瘤生长；也有报道^[27]^表明通过抑制CD47/SIRPα下游SHP-2，可促进巨噬细胞的M1极化，增强巨噬细胞吞噬力，抑制肿瘤生长。本研究结果与上述研究一致，提示三叶青多糖可能通过抑制癌细胞CD47的表达，抑制巨噬细胞表面SIRPα磷酸化而对SHP-1/2募集不足，避免癌细胞的巨噬细胞免疫逃逸，增强巨噬细胞吞噬力，促进机体抗肿瘤免疫反应，从而抑制肿瘤的形成和转移，达到缓解病症的效果。

综上所述，三叶青多糖可有效增强抗肿瘤免疫效应，可能通过抑制SIRP/CD47信号通路，增强巨噬细胞吞噬力，从而促进机体抗肿瘤免疫反应，缓解症状；三叶青多糖高剂量组作用效果最佳。然而，本研究受实验条件限制未采用免疫组化法定位肿瘤组织CD47、SIRPα、p-SIRPα、SHP-1、SHP-2蛋白且未观察阳性细胞率及染色强度，并且三叶青多糖增强抗肿瘤免疫效应的机制研究尚不够完善，未来将设计回复实验证实三叶青多糖对SIRP/CD47信号通路的抑制作用。

**Competing interests**

The authors declare that they have no competing interests.
